# Preferred SH3 Domain Partners of ADAM Metalloproteases Include Shared and ADAM-Specific SH3 Interactions

**DOI:** 10.1371/journal.pone.0121301

**Published:** 2015-03-31

**Authors:** Iivari Kleino, Annika Järviluoma, Jussi Hepojoki, Ari Pekka Huovila, Kalle Saksela

**Affiliations:** 1 Department of Virology, University of Helsinki and Helsinki University Hospital, Helsinki, Finland; 2 Institute of Biosciences and Medical Technology, University of Tampere, Tampere, Finland; Hungarian Academy of Sciences, HUNGARY

## Abstract

A disintegrin and metalloproteinases (ADAMs) constitute a protein family essential for extracellular signaling and regulation of cell adhesion. Catalytic activity of ADAMs and their predicted potential for Src-homology 3 (SH3) domain binding show a strong correlation. Here we present a comprehensive characterization of SH3 binding capacity and preferences of the catalytically active ADAMs 8, 9, 10, 12, 15, 17, and 19. Our results revealed several novel interactions, and also confirmed many previously reported ones. Many of the identified SH3 interaction partners were shared by several ADAMs, whereas some were ADAM-specific. Most of the ADAM-interacting SH3 proteins were adapter proteins or kinases, typically associated with sorting and endocytosis. Novel SH3 interactions revealed in this study include TOCA1 and CIP4 as preferred partners of ADAM8, and RIMBP1 as a partner of ADAM19. Our results suggest that common as well as distinct mechanisms are involved in regulation and execution of ADAM signaling, and provide a useful framework for addressing the pathways that connect ADAMs to normal and aberrant cell behavior.

## Introduction

ADAM-mediated protein ectodomain cleavage—dubbed shedding—provides means to rapidly modify the amount and function of proteins on the cell surface. ADAM substrates range from growth factors and cytokines to receptors and adhesion proteins. Their cleavage regulates or initiates many cellular processes including cytokine and growth factor signaling, cell adhesion, cell migration, and release of intracellular signaling domains from membrane bound precursor proteins. Shedding is thus a central regulatory mechanism of several major signaling pathways involved in cell proliferation, polarization, adhesion, and migration (reviewed in [1,2]).

ADAM17 with over a hundred identified substrates, and ADAM10 with seventy, are the primary sheddases responsible for most of the ADAM-mediated ectodomain shedding characterized so far [1]. Although they share more than half of the substrates, usually shedding by ADAM10 or ADAM17 is initiated by different stimuli [3,4]. In many cases ADAM10 is either constantly shedding or activated by Ca^2+^ influx, whereas ADAM17 can be activated with stimulation of various signaling pathways, including PKC, MAPK, and PDK1, and in some cases involving ADAM17 cytosolic tail phosphorylation. Recently, ADAM17 substrate selectivity was shown to depend on activation of distinct PKC isoforms [5–7]. However, the regulation of activation and substrate selectivity of ADAMs 10 and 17 is still poorly understood [8].

Other ADAM metalloproteases with reported cellular substrates are ADAMs 8, 9, 12, 15, 19, and 28 [1]. Compared to the ADAM10 and ADAM17, they have more restricted substrate repertoires, and can be called secondary sheddases. However, dysregulation of these ADAMs can seriously affect normal cellular physiology. They all are associated with immune and inflammatory disorders [9,10], different types of cancers [11–13], and thus represent attractive candidates for development of therapeutics. For example, silencing of ADAM9 prevents tumor invasiveness *in vitro* and blocks cancer metastasis *in vivo* [14,15].

Compared to ADAM10 and ADAM17, even less is known about the regulation of sheddase activity of the secondary sheddase ADAMs. Different PKC isoforms have been associated with ADAM9 and ADAM12 regulation [16,17], and in some cells stimulation of G-protein coupled receptors induce EGF-like growth factor shedding mediated by ADAM12 or ADAM15 [18,19]. SH3 domains of Eve-1 and Pacsin3 interact with many ADAMs and can regulate at least hb-EGF shedding by ADAM12 [20,21], suggesting that intracellular SH3 domain interactions regulate ectodomain activity of the ADAMs. Interestingly, alternative splicing can profoundly modulate the number and composition of SH3 binding motifs in the ADAM15 cytosolic tail [22–24], and the presence of an alternatively used Src SH3 target motif can increase the capacity of ADAM15 to stimulate fibroblast growth factor receptor-2 shedding [25]. ADAM15 shows aberrant isoform expression in breast cancer and the presence of certain isoforms correlate with poorer relapse free survival [26,27].

Src homology 3 (SH3) domains are small protein interaction modules that recognize proline-rich motifs in their target proteins [28]. They can be found in almost 200 human proteins, often in multiple copies, whereby they regulate numerous cellular signaling pathways, for example via determining the catalytic activity and ligand selectivity of protein kinases [29]. The multiple SH3 domains and/or SH3 binding sites found in many adapter proteins serve to coordinate the assembly of multi-protein signaling complexes, often related to sorting, endocytosis, and actin remodeling, which are all highly relevant cellular functions for ADAM regulation and signaling.

Another important aspect of ADAM tails is their capacity to activate intracellular signaling pathways, which can also be regulated by SH3 interactions. For example binding of the SH3 domain of Src tyrosine kinases or p85α to ADAM12 activates PI3K signaling [30,31], and extracellular cross-linking of ADAM12 induces clusters of invadopodia in a Src and SH3 binding dependent manner [32]. Likewise, SH3-mediated interactions of ADAM15 with Src promote Src/FAK signaling to render chondrocytes and synovial fibroblasts more resistant to apoptosis [33,34], and Src/Erk1/2 signaling can regulate endothelial cell monolayer permeability [35].

Our previous studies have established the value of the human SH3 domain phage display library screening by identifying preferred SH3 partners for known or suspected ligand proteins [36–39]. We have also used this technology to determine the effects of alternative mRNA splicing on SH3 binding selectivity of ADAM15 [24]. In the current study this approach was used to comprehensively characterize the SH3 binding capacity and preferences of catalytically active ADAMs, which was accomplished by subjecting our human SH3 library for exhaustive screens using the intracellular domains of ADAMs 8, 9, 10, 12, 15, 17, and 19.

## Materials and Methods

### Cell Lines and Reagents

Human embryonic kidney (HEK) 293T cells were maintained in Dulbecco modified Eagle medium (DMEM) supplemented with 10% fetal calf serum (FCS), L-glutamine, penicillin, and streptomycin. Transient transfections for ADAM-tail bait productions were done with calcium phosphate as described in Kleino et al. [24] and for co-immunoprecipitations with Fugene 6 according to the manufacturer's instructions (Roche Diagnostics Corporation, Indianapolis, IN). All chemicals for cell culture, cell lysis, and protein works were from Sigma Aldrich (Sigma-Aldrich) if not stated otherwise.

### Construction of Expression and Phagemid Vectors

Biotinylated ADAM-tail proteins were produced by expressing them as fusion proteins with a biotinylation target domain (BTD). For ADAM15i6, the cytosolic tail encoding fragment was subcloned from pGEX-construct used in Kleino et al. 2009 [24] into C-terminal position into pEBB[BTD-mCherry], a pEBB [40] derivative expressing mCherry-BTD fusion BTD being 1–122 residues of the C-terminal biotin acceptor domain of the 1.3S subunit of *Propionibacterium shermanii* derived from the PinPoint Xa-1 vector (Promega). The following construct will express flexible linker SGGDRWSSTGGGR between the mCherry and ADAM15. For production of biotinylated ADAM8, -9, -12, -17, and -19 tails vectors expressing EGFP-BTD-ADAM-tail (eBTD-ADAM-tail) fusion proteins were constructed as follows. For empty vector eBTD fragment of the above mentioned BTD (amino acids 49–112) was inserted into multiple cloning site of pEGFP-C1 (Invitrogen) producing a SGLRSTGGTM linker between the EGFP and BTD. The ADAM-tails encoding DNA fragments were subcloned from corresponding pGEX-ADAM-tail vectors into downstream of BTD producing ERSPEF linker between the BTD and each ADAM tail. The original pGEX ADAM-tail plasmids were constructed by inserting PCR amplified DNAs from image clones with accession numbers BC024214 (ADAM19), BQ708311 (ADAM8), and PCR amplified DNAs derived from cDNAS produced from SK-BR3 or Jurkat RNAs (ADAMs -9, -12, and -17). The ADAM-tails expressed from above mentioned vectors are exactly as follows: ADAM8: HIIVYRKARSRILSRNVAPKTTMGRSNPLFHQAASRVPAKGGAPAPSRGPQELVPTTHPGQPARHPASSVALKRPPPAPPVTVSSPPFPVPVYTRQAPKQVIKPTFAPPVPPVKPGAGAANPGPAEGAVGPKVALKPPIQRKQGAGAPTAP* ADAM9: RDQLWRSYFRKKRSQTYESDGKNQANPSRQPGSVPRHVSPVTPPREVPIYANRFAVPTYAAKQPQQFPSRPPPPQPKVSSQGNLIPARPAPAPPLYSSLT* ADAM10: QICSVHTPSSNPKLPPPKPLPGTLKRRRPPQPIQQPQRQRPRESYQMGHMRR* ADAM12: RKTLIRLLFTNKKTTIEKLRCVRPSRPPRGFQPCQAHLGHLGKGLMRKPPDS YPPKDNPRRLLQCQNVDISRPLNGLNVPQPQSTQRVLPPLHRAPRAPSVPARPLPAKPALRQAQGTCKPNPPQKPLPADPLARTTRLTHALARTPGQWETGLRLAPLRPAPQYPHQVPRSTHTAYIK* ADAM15: MLGASYWYRARLHQRLCQLKGPTCQYRAA QSGPSERPGPPQRALLARGTKQASALSFPAPPSRPLPPDPVSKRLQAELADRPNPPTRPLPADPVVRSPKSQGPAKPPPPRKPLPADPQGRCPSGDLPGPGAGIPPLVVPSRPAPPPPTVSSLYL* ADAM17: HSILVHCVDKKLDKQYESLSLFHPSNVEMLSSMDSASVR IIKPFPAPQTPGRLQPAPVIPSAPAAPKLDHQRMDTIQEDPSTDSHMDEDGFEKDPFPNSSTAAKSFEDLTDHPVTRSEKAASFKLQRQNRGDSKETEC*

GST-SH3 expression vectors were prepared by subcloning SH3 domain sequences from SH3 phagemids described in [24,36] into pGEX-4T-1. Tagged ADAM8, SNX33, and TOCA1 used in co-immunoprecipitations were constructed as follows. Full length ADAM8 from plasmid IMAGE ID: 5324943 was PCR-amplified and inserted in-frame into pEBB derivative containing C-terminal HA-tag. EGFP-SNX33 expression vector was produced by PCR amplifying SNX33 fragment from pEBB-BTD-SNX33 [24] and by inserting it into pEGFP-C1 (Clontech). pEGFP-TOCA1 vector was a gift from Pietro De Camilli.

### Protein Production

Phage screen target-proteins were expressed in and purified from HEK293T cells as follows. 3.5 x 10^6^ HEK293T cells in a 6-well plate were transfected with expression vector for ADAM-tail or control protein. The transfection efficiency was monitored by determining the EGFP or mCherry distribution and levels with inverted fluorescence microscope (Olympus). 36 h post transfection the cells were collected into ice cold PBG-buffer [PBS solution with 10% glycerol] supplemented with Complete protease inhibitor cocktail (Roche). Cell lysis and release of proteins was accomplished by sonication of the cell suspension for 7 cycles of 4 seconds on/3 seconds off with 40% amplitude in Sonopuls HD 3200 sonicator (Bandelin). Lysates were cleared by centrifugation at 12.000 x g for 30 min and stored in -20°C. GST-SH3 proteins used in probing of CelluSpot peptide arrays were produced in pGEX-SH3 transformed BL21 *E*.*coli* and purified with glutathione 4B beads according to standard batch purification protocol in manufacturers manual (GE Healthcare).

### Phage Libraries and Screening

For the 117 independent phage screens done in this work, 34 custom SH3 phage libraries (S1 Table) were prepared from M13 derived phagemid vectors described in [36] by using methods described in [36,41]. To avoid library based bias, at least two independently prepared libraries with the same SH3-domain compositions were prepared and screened at each selection stage. Fresh phage libraries were filtered with 0.22 μm filter and stored at +4°C and used within 4 weeks from the preparation. We have not observed any weakening in library performance during that time. If strong ADAM-binders were identified from the library, a new reduced library lacking the identified SH3-domain phages were prepared. Differing from the original full SH3 phage library [36], all libraries used in this study lacked SH3 phages of Crk(II) and CrkL(II) as we have previously observed these atypical SH3 domains to be prone for selection without true affinity to diverse targets and hence causing false positives.

SH3 phage screening was done essentially as in [24,36] except that instead of a plastic surface, the target proteins were immobilized onto streptavidin magnetic beads. The following describes the differences to previous protocols [24,36]. HEK293T cell lysates containing biotin ADAM-tail proteins were transferred on 7 μl of M-280 streptavidin Dynabead suspension (Invitrogen) in 1.5 ml Eppendorf tubes. Streptavidin beads were allowed to attach the biotinylated proteins in the lysates in room temperature (RT) in slow rotation for 1 h followed by 4 washes with 1.5 ml of the PBG. Finally the beads with captured target proteins were resuspended into 50 μl of PBST (PBS + 0.05% Tween 20). For each screen 400 μl of the phage library was blocked with 400 μl of 4% milk in PBST and then added on the target protein-beads and incubated for 2 h in rotation in RT. After capture, the beads were washed four times with PBST. To prevent tube carried contamination the bead-target mix was transferred into fresh tube between the third and fourth washes. After final wash the bait-phage mix was suspended into 100 μl of sterile LB medium, and 50 μl of this was used to infect *E*.*coli* as described in [36]. The identity of the SH3 domains of the selected phages was determined as described in [24]. EGFP, mCherry, BTD, or endogenous bead bound material from 293T captured with M-280 beads was found negative in selecting SH3 domain phages as indicated by small number of retained phages and unbiased set of phages identified amongst retained ones (79 different SH3s out of 119 phages).

### CelluSpot Peptide Arrays Probing

Synthesis and probing of CelluSpot arrays were done essentially as described in [24]. In current study, each peptide array slide contained three separate sub-arrays of peptides. To prevent oversaturation and to see differences better with weaker binders different concentrations of GST-SH3 domains were used to probe the slides as follows: 20 μg/ml, Hck, Eps8L1, Nck(I), SNX9, TOCA1, RIMBP1(III); 15 μg/ml, Tec, RIMBP2(III), RIMBP3(III), formin binding protein 17 (FBP17), 10 μg/ml Tks5(I), Tks5(V), intersectin1(III), intersectin2(III), SNX18, Lyn, Fyn, Itk, Btk, Lck, CIP4, NCF1(I), Grb2(II), AHI1, OSTF1; 5 μg/ml SNX33, Src, Grb2(I), nephrocystin, and 2 μg/ml p85α. These concentrations were empirically chosen to maximally differentiate the peptide binding signal intensities, and to achieve an optimal signal to noise ratio for each SH3 domain probe.

The bound GST-SH3 domains were detected with IRDYE-680 labeled anti-GST (Li-cor Biosciences) at 1:5000 dilution and the signals from probed slides were scanned in Li-cor Odyssey Near Infrared imaging system (Li-cor Biosciences). Individual spot signals from slide images were quantified by Odyssey software (Li-cor Biosciences). Each ADAM-peptide-SH3 signal was averaged from three spots, which were first subtracted with slide specific background. Average signal from 33 empty spots increased with 2x standard deviations of empty spots was used as background. To exclude non-specific binding of GST or anti-GST-antibodies to peptides the slides were also probed with varying concentrations GST-only and signals from those were compared to patterns from ADAM-binding SH3 domains. Each SH3-domain probing was normalized to strongest signal showing in each column as 2. Data was analyzed with Cluster 3 software (Eisen, eisen@rana.lbl.cov and de Hoon, mdehoon@gsc.riken.jp, http://bonsai.hgc.jp/~mdehoon/software/cluster/). The peptidearray image was produced in Java TreeView (Alok, alokito@users.sourceforge.net, http://jtreeview.sourceforge.net).

### Antibodies, Western Blotting and Immunoprecipitation

Mouse monoclonal antibody recognizing the HA-epitope (F-7) and rabbit polyclonal antibody to GFP (sc-8334) were purchased from Santa Cruz Biotechnology (Santa Cruz, CA). The mouse monoclonal antibody to α-tubulin (T6199) was from Sigma-Aldrich Corp. (St. Louis, MO). When Western blotting tissue culture cells were lysed in KEB lysis buffer [137 mM NaCl, 50 mM Tris/HCl [pH 8], 2 mM EDTA, 0.5% Nonidet P-40, and protease inhibitors]. Fifty micrograms of total proteins were analyzed by 8% sodium dodecyl sulfate-polyacrylamide gel electrophoresis (SDS-PAGE) and blotted according to standard protocols.

For detection of association between ADAM8 and SNX33 or TOCA-1, HEK293T cells were transfected with ADAM8-HA, EGFP-TOCA-1, and EGFP-SNX33 expression vectors, lysed with the KEB lysis buffer. Cleared cell extracts (600 μg) were incubated with anti-HA mouse monoclonal antibody for 2 hours at +4°C. Immunocomplexes were coupled to protein G Sepharose beads (Dynabeads Protein G, Invitrogen, San Diego, CA) for additional 2 hours at 4°C and washed 3 times with the lysis buffer. The immunoprecipitates were boiled in Laemmli sample buffer and subjected to SDS-PAGE and Western blotting analysis.

## Results

### Phage library profiling of SH3 binding capacity and selectivity of ADAM tails

**Fig 1:**
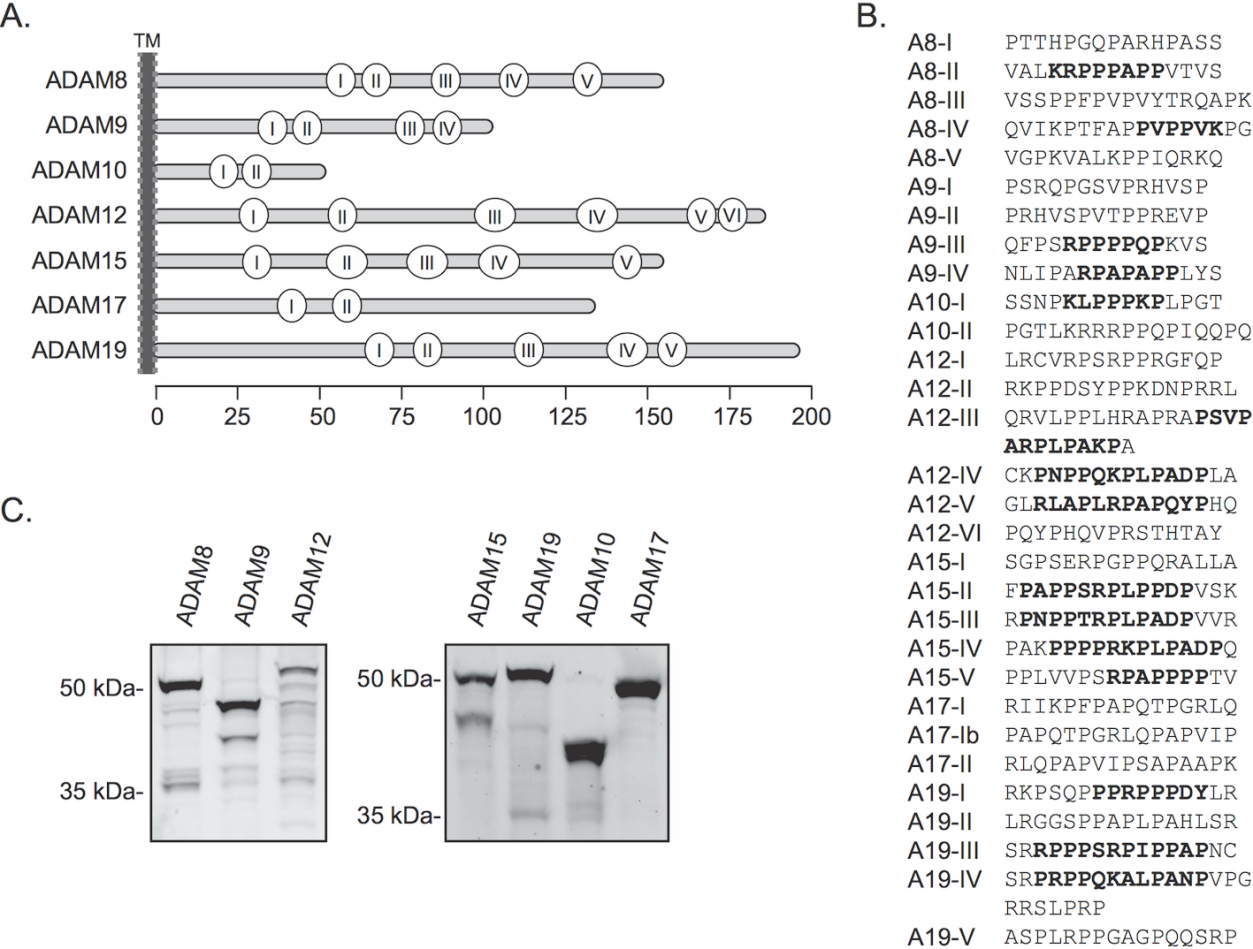
Cytosolic tails of ADAM-metalloproteases and their candidate SH3 binding motifs. **A.** Schematic presentation of ADAM cytosolic tails with the location of the candidate SH3-binding proline clusters indicated by circles with roman numerals counting from the transmembrane region to the carboxy terminus. The scale bar indicates distance in amino acid residues. **B.** Potential SH3 binding sequences within the proline clusters shown in A. Established SH3 target motifs (+ΦPxxP, PxΦPx+, PxxDY, where + is K or R and Φ is a hydrophobic residue) occurring individually or in clusters where they partly overlap each other are indicated in bold. **C.** Western blotting analysis of the ADAM tails expressed as biotinylated fusion proteins in human 293T cells for use as affinity baits in SH3 domain library screening.

To identify human ADAMs with SH3 binding potential we first examined their cytosolic domains for the presence of canonical SH3 target motifs or other proline-rich sequences suggestive of SH3 binding (see [28]). With the exception of ADAM28 and ADAM33 one or more candidate SH3-binding sites were evident in all of the 9 ADAMs with established catalytic activity (Fig 1A and 1B). By contrast, with the exception of ADAM22, no potential SH3 target sites could be identified in the 11 other ADAMs.

The intracellular domains of ADAMs 8, 9, 10, 12, 15, 17, and 19 were used as affinity ligands for screening of our human SH3 domain phage-display library. These ADAM tails were expressed in human 293T cells as fusion proteins (Fig 1C) containing a biotinylation target domain (BTD) and a fluorescent domain (Materials and methods), and captured on paramagnetic streptavidin-coated beads for phage library panning. The overall success of this strategy was found superior compared to plastic well passively coated with ADAM-tails expressed as GST fusion proteins in *E*. *coli* (data not shown).

**Fig 2:**
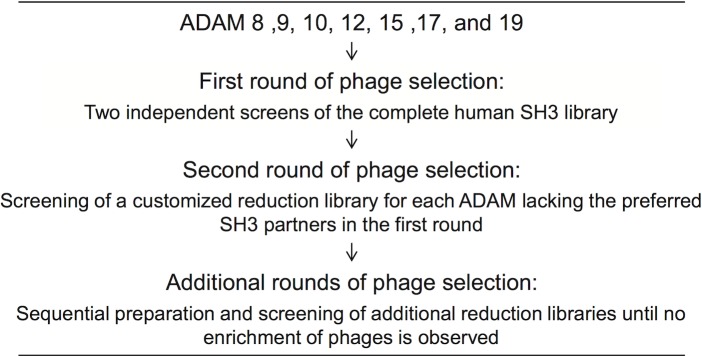
Outline of phage library screening. A sequential strategy was used for screening of complete as well as subsequent rationally customized SH3 display libraries in order to exhaustively assess potential SH3 partners of the indicated ADAMs.

All ADAM-tails were used at least twice to screen independently prepared versions of the complete SH3 phage library. To better identify also the SH3 domains binding these ADAMs with a lower affinity additional libraries were sequentially constructed and screened as outlined in Fig 2 and detailed in S1 Table. SH3 phages that dominated among the clones selected by ADAMs 8, 9, 12, 15, and 19 in the prior selection experiment were excluded from these customized “reduction libraries” used for further affinity panning.

The overall enrichment of phages from the complete SH3 library (i.e. apparent SH3 binding capacity) varied greatly between the different ADAMs. As shown in Table 1, ADAMs 8, 9 and 15 supported robust SH3 phage enrichment, ADAMs 12, and 19 also showed strong albeit less intense SH3 phage binding capacity, whereas phage enrichment by ADAMs 10 and 17 was modest.

**Table 1:** Key statistics of phage library selection experiments.

	**A8**	**A9**	**A10**	**A12**	**A15**	**A17**	**A19**
Phage enrichment in the first round of library screening	3000	>10,000	50	200	10,000	5	500
Number of customized reduction libraries prepared and screened	5	4	0	3	6	0	4
Total number of SH3 clones identified by sequencing	332	142	190	384	172	47	203
Total number of selected SH3 domains	8	7	0	8	14	0	14

Accordingly, sequencing of a large number (n = 237) of phage clones obtained from panning with ADAM10 or ADAM17 indicated no obvious selectivity towards any SH3 domain. By contrast, enrichment of phages by ADAMs 8, 9, 12, 15, and 19 was in all cases also associated with an apparent SH3 binding selectivity. The two SH3 domains most frequently selected from the complete SH3 library represented 94% of all sequenced phage clones for ADAM9 as well as for ADAM15, 80% for ADAM19, 72% for ADAM8, and 47% for ADAM12. The identities of these dominant SH3 domains differed between the ADAMs, although sorting nexin 33 (SNX33) was found to be the preferred partner for three different ADAMs (ADAM8, ADAM 9, and ADAM15).

Further screening of reduction libraries customized for each ADAM led to identification of 4 to 9 highly selected SH3 domains for each of these ADAMs. These SH3 domains are classified as “dominant” or “strongly selected” in Table 2, which also lists SH3 domains that were modestly but reproducibly selected (classified as “significantly selected”).

**Table 2:** SH3 domain clones selected by intracellular tails of different ADAMs.

	**ADAM8**	**ADAM9**	**ADAM12**	**ADAM15**	**ADAM19**
**Dominant**	SNX33	SNX33	Src*	SNX33*	Tks5(I)
	TOCA1	SNX9*	Nephrocystin		
**Strongly selected**	CIP4	Tec	Lyn*	Tks5(V)*	Src
	SNX9	SNX18	Tks5(V)*	Src*	RIMBP1(III)
	Tec		Hck*	SNX9*	Eps8L1
	Src		AHI1	Nephrocystin*	NCF1(I)
				Hck*	
				Tec	
				Lyn*	
				Tks5(I)*	
**Significantly enriched**	NCF1(I)	NCF1(I)	SNX33	p85α*	Tec
	OSTF1	Lyn	SNX9	Btk	Lyn
		ArgBP2(II)		NCF1(I)*	p85α
				OSTF1	Nephrocystin
				intersectin1(III)	SNX33
					TOCA1
					SNX9*
					Tks5(V)*
					Hck

SH3 domains repeatedly enriched by affinity screening from the complete and subsequently constructed ADAM-specific reduction libraries. SH3 interactions reported previously are indicated with an asterisk.

As discussed later, the ADAM-targeted SH3 domains were most often found in proteins involved in membrane trafficking, and included some previously known ADAM partners (see Table 2) as well as novel interactions such as binding of ADAM8 to transducer of Cdc42-dependent actin assembly-1 (TOCA1) SH3 and binding of ADAM19 to RIMBP1(III) SH3. For proteins containing multiple SH3 domains, the Roman number in parentheses indicates the SH3 domain in question (counting from the N-terminus).

### Mapping of SH3 target motifs by peptide arrays

**Fig 3:**
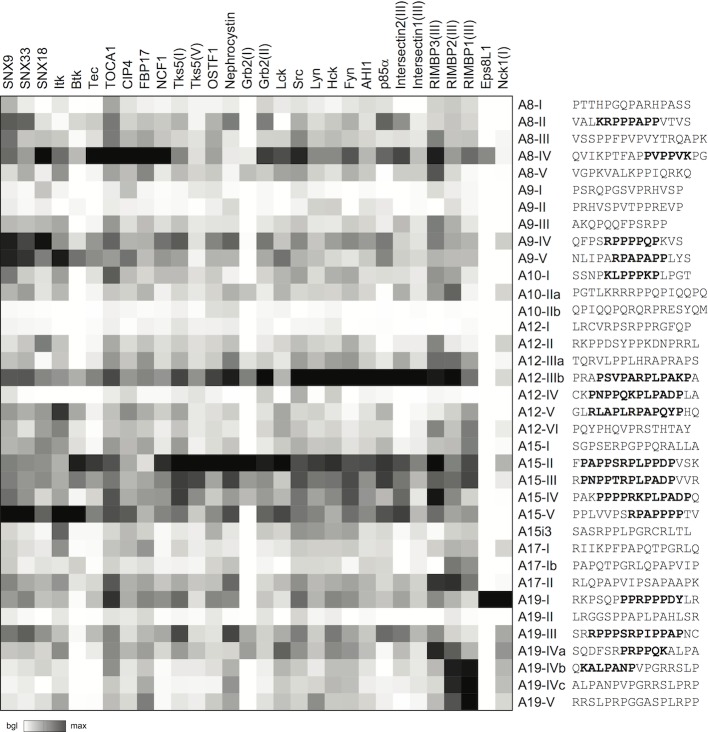
Summary of the peptide array data for identification of functional SH3 binding sites in the ADAM tails. The indicated peptides corresponding to the proline-cluster numbering in Fig 1A were printed in triplicate on arrays slides, and probed with the indicated SH3 domains. Signals of the triplicate spots were averaged, and these values normalized to the average of the peptide giving the strongest signal in each SH3 probing. Shown is a heat map based on these normalized values where white indicates background level binding, and black the strongest peptide binding for each SH3 domain.

Some, but not all, of the SH3 binding specificity and affinity determinants can be defined by short and linear peptide motifs. In order to map the SH3 target motifs within the intracellular tails of ADAM 8, 9, 10, 12, 15, 17, and 19, as well as to examine the overall correlation between our phage-library data and binding of the selected SH3 domains to defined proline-rich motifs in these ADAMs, a peptide array was designed (Fig 3).

Peptides ranging from 14 to 17 amino acids in length corresponding to the putative SH3 target sites were synthesized and spotted on slides. The slides were probed with 30 different SH3 domains expressed as recombinant GST fusion proteins and the binding was detected with a fluorescent anti-GST antibody. These SH3 domains were chosen to cover as many as possible of the interactions identified by phage display (when a soluble SH3 probe could be expressed), but also included some related SH3 domains (such as Src family members), as well as SH3 domains not identified in our screen but described in the literature as ADAM partners.

The results are summarized as a heat map in Fig 3, showing relative binding of each SH3 domain to the test set of 36 ADAM peptides. As apparent from Fig 3, distinct specificities could be observed, and all SH3 domains showed unique peptide array binding profiles. As expected, the binding profiles of related SH3 domains, such as members of the Src-family kinases (SFK) were more similar to each other than—for example—to SNXs. On the other hand, a preference for canonical motifs typical for SFK was shared by many SH3 domains, such as nephrocystin, AHI1, OSTF1, intersectin1(III), and Grb2(II).

All SH3 domains classified in Table 2 as dominant or strongly selected ADAM partners were among the strongest (top six) binders for at least one peptide from the ADAM protein that selected them from the phage library. On the other hand, certain ADAM-peptides, like A12-IIIb and A15-II, bound robustly to the majority of the tested SH3 domains, including many that were not selected by ADAM12 or ADAM15 from the phage library.

Despite a general correlation between these two data sets it was also clear that the binding preferences of the complete ADAM tails revealed by the SH3 library screening could not be predicted by the peptide array data. Nevertheless, in most cases the peptide array was informative in identifying the peptide region serving as the key docking site for each ADAM-binding SH3 domain.

The number of binding sites and their contribution to SH3 binding varied between the ADAMs. For example, a single (out of the six predicted) SH3 target site in ADAM12 (A12-IIIb) accounted for all of the strongest interactions by the eight ADAM12-selected SH3 domains. By contrast, four out of five different target sites in ADAM15 bound with distinct specificities to the panel of SH3 domains selected by ADAM15. On the other hand, two of the four target motifs in ADAM9 (A9-IIIb and A9-IV) bound with similar strength but high specificity to SH3 domain partners of ADAM9, namely Tec and SNXs.

Most of the ADAM peptides that showed strong SH3 binding contained canonical class I SH3 binding motifs. Notable exceptions were the class II binding site in ADAM8 (A8-IV) that bound strongly to multiple SH3 domains, and the two overlapping peptides of ADAM19 (A19-IVc and A19-V) that did not contain a known consensus motif, but bound tightly to the third SH3 domains of RIMBP1 and RIMP2.

### ADAM8 interacts with TOCA1 and SNX33 in transfected human cells

The preferred SH3 partners for ADAM8 found in our screens were TOCA1 and SNX33. Moreover, when these two were excluded from the phage library ADAM8 preferentially selected the SH3 domains of Cdc42 interacting protein 4 (CIP4) and SNX9, which are the closest relatives of TOCA1 and SNX33. TOCA1 and CIP4 were specifically selected only by ADAM8, and have not been previously reported to interact with any ADAM. On the other hand, SNXs were found to be preferred SH3 partners of many ADAMs, and have already are been reported to interact with ADAMs 9, and 15. However, ADAM8 has not been previously implicated as an SNX-ligand.

**Fig 4:**
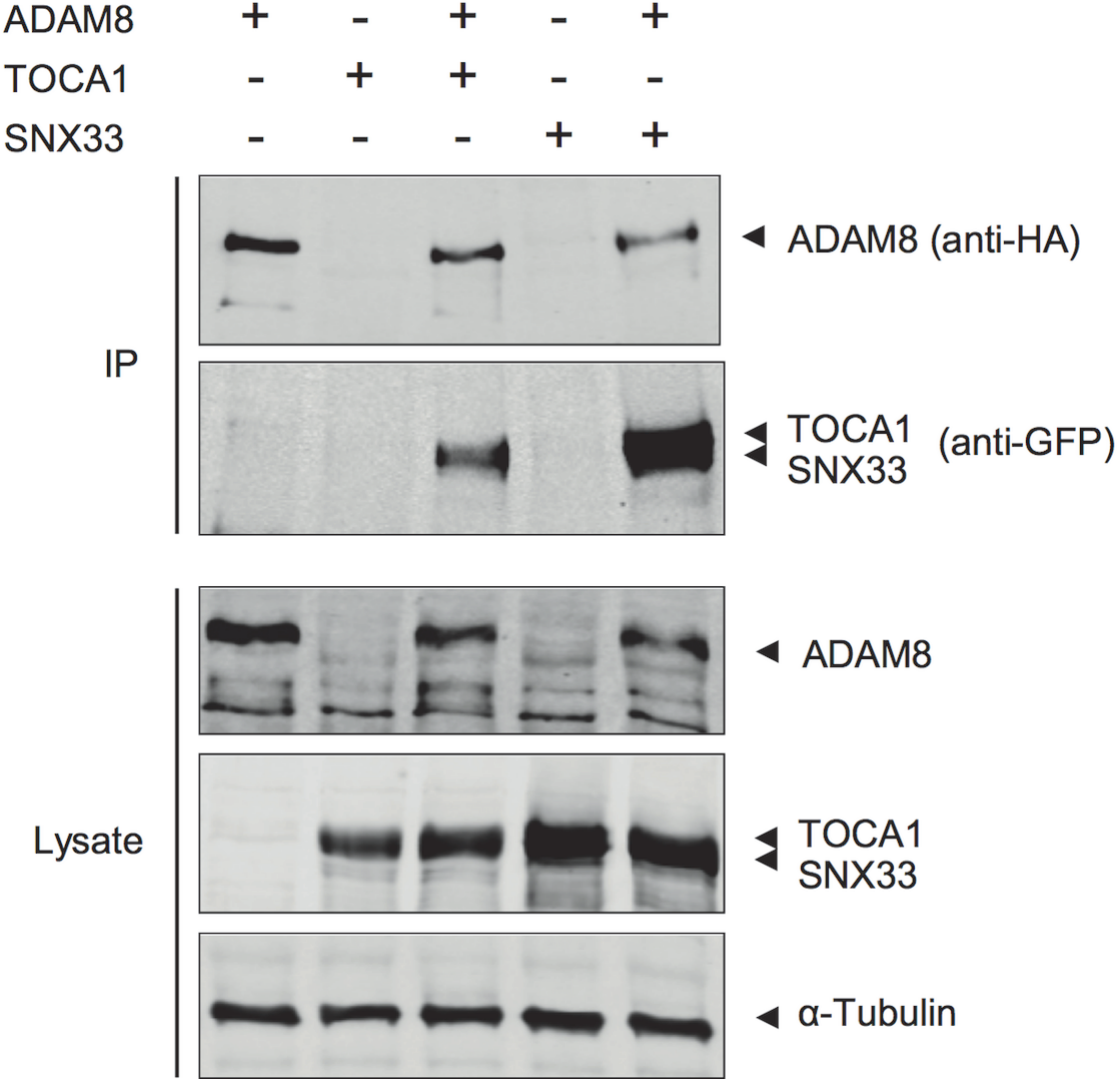
ADAM8 interacts with TOCA1 and SNX33 in human cells. SNX33 and TOCA1 were expressed as EGFP-fusion proteins by transiently transfected 293T cells alone or with HA-tagged ADAM8 as indicated on top of the figure. The presence of ADAM8 itself (top panel) or TOCA1 or SNX33 that was associated with it (second panel from top) in anti-HA immunoprecipitates (IP) from the lysates of these cells was examined by Western blotting. Similar total levels of ADAM8, TOCA1, and SNX33 between the transfected cells were confirmed by a Western blotting analysis of the unselected lysates (Lysate). Blotting for the endogenous α-tubulin in these lysates was included as an additional loading control.

To extend our *in vitro* binding results, we decided to study interactions of ADAM8 with TOCA1 and SNX33 using full-length native proteins expressed in human cells. Expression vectors for epitope- or GFP-tagged version of ADAM8, TOCA1, and SNX33 were co-transfected into HEK 293T cells, and their interactions studied by co-immunoprecipitation. As shown in Fig 4, TOCA1 and SNX33 could be readily detected from anti-HA immunocomplexes from lysates of ADAM8-HA transfected cells, but not from control cell lysates expressing TOCA1 and SNX33 only.

## Discussion

ADAMs are attractive targets for therapeutic intervention in many pathological conditions, including various types of cancer [11–13]. Presence of putative SH3 domain binding motifs is a prominent feature of the cytosolic tails of ADAMs with catalytic activity. In this study we used a comprehensive human SH3 domain phage display library in combination with peptide array technology to systematically characterize the SH3 binding capacity and preferences of ADAMs 8, 9, 10, 12, 15, 17, and 19.

The peptide array results helped to characterize the core binding motifs in the ADAMs for their preferred SH3 domain partners, and showed a general correlation with the phage display data regarding the SH3 partnering specificity of the ADAMs. However, the peptide data could not be used to predict the SH3 interactions of the complete ADAM tails revealed by the phage library screening. Thus, binding determinants beyond individual the proline-rich motifs clearly provide an important contribution to the SH3 domain selectivity of the ADAM tails. Such determinants could include contacts with regions in the cognate SH3 domain outside of its proline-rich peptide binding groove, or residues that can influence the conformation of the proline-rich motifs to increase their SH3 binding affinity and specificity.

Several reports have established that the cytosolic tails of ADAM9, 12, 15, and 19 bind a number of different SH3 domains (see review by [1]). Thirteen of the 24 highly preferred SH3 domain interactions that we identified have been described in previous studies, demonstrating the power of our approach. Many of the 13 “re-discovered” ADAM interactions involve the SH3 domains of Tks5 or a member of the SFK or SNX protein families, which we found to be highly redundant partners of ADAMs considered as the secondary sheddases especially (i.e. proteolytically active ADAMs other than ADAM10 and ADAM17).

Despite the apparent in vitro promiscuity in binding of SFK/Tks SH3 domains by ADAMs, these interactions are involved in guiding of complex and highly regulated cellular processes, as exemplified by the role of ADAM15 in Src/Erk1/2 signaling in endothelial cells [35], Src/FAK in chondrocytes [34], and ADAM12 signaling via Src/Tks5 in migrating cells [32,42].

Tks5 is a Src substrate that regulates the formation of podosomes and invadopodia via ADAM12 [32,43], and serves as a regulatory component of the reactive oxygen species (ROS) producing NADPH oxidase (NOX) complex [44]. Interestingly, we found that ADAMs differ in their strategies to interact with Tks5 via its multiple SH3 domains. ADAM12 and ADAM15 both bind strongly to Tks5(V), whereas ADAM15 also binds to Tks5(I), which is the principal SH3 target of ADAM8 and ADAM19.

The ADAMs that bound to Tks5(I) also selected NCF1(I) as one of their preferred SH3 partners. NCF1 (alias p47phox or NOXO2) is a regulatory subunit of phagocyte NAPDH oxidase, which together with Tks5 belongs to the NAPDH complex organizer superfamily. Indeed, the N-terminal regions of Tks5 and NCF1 comprise of a PX and two SH3 domains that are very similar even at the sequence level. Taken together our observations suggest a general role for ADAMs in spatial coordination of cellular ROS production and its connection to tyrosine phosphorylation. It is interesting to note that NCF1 protein is using both of its SH3 domains to sandwich proline-rich ligand peptides between them [45]. It remains to be investigated whether Tks5 uses a similar double SH3 strategy in binding to ADAMs or peptides from other ligand proteins.

In addition to the Src-family tyrosine kinases, ADAMs were also found to interact significantly with SH3 domains of the Tec-family tyrosine kinases [46], which have previously not been identified as ADAM binding partners. Tec was selected by ADAMs 8, 9, 15, and 19, while ADAM15 also bound Btk. ADAMs 8, 15, and 19 are constitutively expressed or their expression is induced after stimulation in many hematopoietic lineages, especially in different macrophages (see immgen.org for expression profiles). In addition to their roles in lymphocyte activation, Tec family kinases are also expressed in macrophages and other cells of myeloid origin [47]. Considering the close functional connections between the Tec and SFK kinase families in hematopoietic cell signaling [48], it is tempting to speculate that their coordinated interactions with ADAMs might help to regulate the development and function of cells in the immune system.

Our current results confirm and further emphasize the role of SNX9 and SNX33 as prominent cellular interaction partners of ADAM9 and ADAM15 as previously reported by us and others [24,36,49]. In addition, we now found that SNX18, the third member of SH3-PX-BAR subfamily of SNXs [50] is also a preferred binder of ADAM9 and SNX33 is very strong binder of ADAM8. However, unlike ADAM9 and ADAM15, the cytosolic tail of ADAM8 does not contain the RxAPxxP sequence, a high affinity binding motif of the SNX9 SH3 domain [51]. Instead, ADAM8 uses two different proline rich SH3 binding sequences for SNX binding. Members of the SH3-PX-BAR subfamily of SNXs are involved in cellular protein sorting, endocytosis, and dorsal circular ruffle formation, and are required for successful mitosis [50,52]. When considering the cell biology of ADAMs, it may not be a co-incidence that SNXs and NADPH complex organizer proteins (including Tks and NCF1) are the two predominant protein families that utilize a PX domain (phox domain) as their phosphoinositide-binding membrane targeting module.

The functional role and significance of the SNX interactions by ADAMs are not known. However, based on the established functions of SNXs in clathrin-dependent endocytosis and intracellular protein sorting it is logical to assume that SNXs might regulate subcellular targeting and availability of ADAMs on the plasma membrane. Moreover, the capacity of the multiple ADAM SH3-binding motifs to connect additional SH3-containing proteins with SNXs suggest that beyond their sheddase activity ADAMs might have a general role as membrane anchored adaptor proteins in cellular signaling and trafficking. In support of this idea, such function has been described for ADAM12 in subcellular targeting of TGFβ-type II receptor after TGFβ binding [53].

Like the SNXs, nephrocystin, another SH3-protein previously reported as an avid ADAM15 interacting protein [24,36], was identified as a dominant binding partner of ADAM12 in the current study, and was also a frequently selected SH3 partner of ADAM19. In agreement, in peptide array tests at least one peptide in ADAM12, ADAM15, and ADAM19, but not in other ADAMs, gave an intense signal when probed with nephrocystin SH3 domain. The avidly binding peptides ADAM12 and ADAM15 contain the RxLPxxP sequence that we have previously identified as a high affinity binding motif for nephrocystin SH3 [24], while ADAM19 carries a related RxIPxxP motif.

In cells nephrocystin localizes to the base of the cilia [54], to focal adhesions, and to adherence junctions [55]. Furthermore, it has been reported to interact with p130Cas, an important Src substrate in transformed and migrating cells [55–57]. Mutations in nephrocystin cause the most common form of childhood cystic kidney disease, nephronophthisis, by affecting ciliary sorting/signaling and cell polarization [55]. Interestingly, ADAM12, a strong nephrocystin binder, also selected the SH3 domain of another ciliary disease-associated protein AHI1/Jouberin [58]. Thus, understanding how the interactions of ADAM12/15 with nephrocystin and/or AHI1 might be involved in regulation of cell polarization, adhesion, migration, or in outside-in signaling through these ADAMs seem like promising areas for further investigation.

Until now, no SH3 interactions have been described for ADAM8. Remarkably, we found that ADAM8 bound strongly and almost uniquely to two closely related proteins TOCA1 and CIP4. Although the focus of this study was to comprehensively profile SH3 binding capacity and preferences of the ADAM family, initial steps towards characterization of the cell biology were taken in the case of ADAM8. In this regard we could show that binding of ADAM8 to SNX33 and TOCA1 is not limited to our initial study systems involving isolated SH3 domains and a cytosolic ADAM8 tail, but could be readily verified with corresponding full-length proteins in human cells.

Like the SH3-PX-BAR subfamily of SNXs, TOCA1 and CIP4 contain a combination of an SH3 domain and a membrane curvature sensing/inducing BAR domain, and they induce actin polymerization associated with vesicle trafficking and protein sorting [59]. Many proteins with a BAR or F-BAR domain also have an SH3 domains. Recently a potentially general autoinhibitory mechanism controlling the membrane deforming activity of such proteins was described for syndapin 1 [60]. This involves the release of an inactive clamped conformation by binding the SH3 domain of syndapin 1 to external proline-rich ligands. Thus, it is reasonable to suggest that CIP4 and TOCA1 are also regulated by a similar SH3-mediated autoinhibitory mechanism, and that binding to ADAM8 would trigger their membrane remodeling activity.

Interestingly, ADAM8, TOCA1, and CIP4 are all associated with breast cancer metastasis [61,62] and invadopodia formation. Thus, the interaction of ADAM8 with TOCA1, and/or CIP4 could be worth a closer look as a potential mechanism driving breast cancer progression.

Further examples of previously unreported and highly ADAM-specific interactions identified in this study include the binding of ADAM19 to the SH3 domains of Eps8L1 and Rim binding protein 1 (RIMBP1; also known as PRAX-1). Both Eps8L1 and RIMBP1 serve complex, but so far incompletely understood, roles as adaptor proteins in cellular signaling, making it premature to propose detailed hypotheses regarding their possible contributions to ADAM19 biology.

Eps8L1 is a member of the Eps8-family of protein involved in regulation of actin remodeling via small GTPases [63], and contains an SH3 domain with atypical binding preference towards a PxxDY motif [64]. Indeed, such a motif is found in ADAM19, and bound strongly to Eps8L1 SH3 domain in our peptide array, albeit less strongly than CD3ε peptides (data not shown).

RIMBP1 is a multi-domain protein with three SH3 domains and FN type III repeats [65]. It serves to couple Rab3-containing vesicles to voltage gated calcium channels in neuronal synapses. ADAM19 is also expressed in neurons and regulates neuromuscular junction development together with EphA4 [66] suggesting a possible connection to synaptic regulation along with RIMBP1. Non-neural cells also express RIMBP1, which interacts with the peripheral benzodiazepine receptor and shows a cytoplasmic localization that includes vesicular structures [67]. ADAM19 predominantly localizes to Golgi membranes and to intracellular vesicles [68], suggesting that these proteins could interact in cells.

Specific binding of the third SH3 domain of RIMBP1 (and RIMB2) to ADAM19 was detected in our peptide array studies. The core SH3-binding region in ADAM19 contains several proline and arginine residues but lacks a known SH3 binding motif, thus apparently harboring yet another atypical SH3 target sequence.

The identification of several previously known ADAM-interacting proteins in our SH3 library screens provides good support for the relevance of the novel ADAM-binding partners that we discovered. On the other hand, SH3 domains encoded by a number of previously reported ADAM-associated proteins were not consistently or at all encountered in our phage display screens. Such missed SH3 domains include those of PACSIN2, PACSIN3, and endophilin A1 (reported to bind to ADAMs 9, 10, 12, 13, 15, and 19, [21,49,69], Grb2 and Nck (reported to bind to ADAM15, [27,70], Eve-1 (reported to bind to ADAMs 9, 10, 12, 15, and 17 [20]), and SAP97 (reported to bind to ADAM10 [71].

Of note, we failed to identify any SH3 domains that would be significantly selected from our phage library by the major sheddases ADAM10 and ADAM17. As discussed above, Eve-1 has been reported as a potential SH3-containing ligand for ADAM10 and ADAM17, whereas ADAM10 has been reported to bind to the SH3 domains of SAP97 and Pacsin3.

The reasons why these SH3 domains were not identified as significant binding partners in our ADAM screens are probably heterogeneous. Significant enrichment of a specific SH3 clone from this phage library requires a threshold binding affinity that may be higher than what weak but yet biologically relevant SH3-ligand complexes can provide. In their natural cellular context such weak interactions may be stabilized by additional protein contacts, in some cases provided by extra SH3 domains in the same protein. It is also important to note that using the IUPred algorithm [72] all the ADAM-tails used in this study can be predicted to have a high likelihood of being intrinsically disordered protein domains. It is therefore possible, if not likely, that interactions with other proteins or plasma membrane lipids may induce novel conformations in these tails (as proposed for ADAM10 by Deng et al [73]), and these in turn might serve as attractive SH3 binding structures that are lacking in our bait proteins despite the fact that they were expressed and purified from human 293T cells.

Moreover, as most ADAMs contain several potential SH3 binding sites, it is noteworthy that instead of individual SH3 domains larger protein fragments or full-length multi-SH3 proteins were used in most of these previous studies (Eve-1 has five, and Nck and Grb2 both two SH3 domains). Thus, these interactions may depend on combinatorial binding of more than one SH3 domain to the same ADAM tail. On the other hand, it should also be noted some of the previously reported interactions may not be SH3-mediated. For example, SAP97 is a member of the MAGUK family, where the SH3 domain is disjoint by pseudo-kinase domain, and therefore unlikely to bind to proline-rich ligands like the ADAM10 tail [74].

It should also be noted that SH3 domains of some of these previously reported ADAM-partners were in fact identified among the ADAM-selected clones that we sequenced, but at such a low frequency that did not meet our criteria of significance. Specifically, SH3 domains of pacsins, endophilins, Grb2, and intersectins were occasionally encountered in our ADAM-screens. However, given the nature of this phage library-based discovery system the relevance of such infrequent hits cannot be ruled out.

Another laboratory [75] recently reported the identification of ADAM10-binding partners using a commercially available version of our SH3 domain library. They used a bacterially expressed version of the intracellular ADAM10 tail as a bait, and reported it to bind as many as 38 different SH3 domains. However, analogously to our studies the overall phage enrichment over negative control appeared to be low, and none of the individual SH3 domains were detected at a high frequency (in fact most were observed only once). Nevertheless, it is interesting to note that the most commonly encountered SH3 domain (2,7% of sequenced clones) was endophilin-A2. Other SH3 domains that were detected more than twice among the 291 sequenced clones selected by GST-ADAM10 (but not GST) were the SFK family member Lck and the palmitoyltransferase ZDHHC6. Our results provide support for these rare hits, as ZDHHC6 and endophilin-A2 (or to be precise the highly homologous SH3 of the endophilin-A2-related pseudogene SH3GL1P2) were repeatedly but infrequently (ZDHHC6 3% and SH3GL1P2 2%) observed in our screens with ADAM10.

Considering that ADAM10 and 17 are central regulators of protein shedding, further studies to identify their relevant SH3 partners are clearly warranted. However, unlike many other ADAMs, they do not show robust and specific affinity for towards any individual single SH3 domain. Therefore, functional rather than affinity-based approaches should be considered to address this question.

In summary, our current study provides a comprehensive survey of the SH3 partners of ADAM sheddases, thereby confirming many previously reported interactions and revealing several new ones. We found that the principal SH3 partners are typically shared by several ADAMs, but also discovered robust ADAM-specific SH3 interactions. The highly shared interaction partners included Src-family kinases and adaptor proteins involved in regulation of actin dynamics and ROS production. More specifically, a defining feature of ADAMs that emerged from our studies was their ubiquitous binding to BAR and SH3 domain-containing proteins associated in actin regulation, protein sorting, vesicle transport, and endocytosis. In addition to two families of such proteins, namely SNXs and NADPH-organizers (which also contain a PX domain) that dominated our interaction screens, a third class of BAR and SH3-containing proteins, namely the PACSIN/endophilin family, has also been reported to bind to ADAMs [21,49,69,75]. SH3 domains from this family were also encountered in our screens, albeit at an inconclusively low frequency.

To generalize our findings, in comparison to other ADAMs, ADAM8 and ADAM9 appeared to be the most dedicated to binding to SH3 domains of sorting proteins, whereas ADAM12 is more specialized to interacting with signal transducing proteins. Selection of TOCA1 and CIP4 as its preferred SH3 partners was a notable and specific property of ADAM8. ADAM19 resembled ADAM12 in its SH3 binding profile, but bound to Tks5 and SFKs less avidly, and was unique in its binding to the scaffolding protein RIMBP1. ADAM15 showed the broadest spectrum of binding to many different SH3 protein families. This can be explained by the multiple ADAM15 isoforms generated via alternative mRNA splicing, which have more unique and restricted SH3 binding potential [24].

Together our data provide an improved overall view of the protein interactomes recruited by ADAMs via their multi SH3 binding site-containing intracellular tails. Detailed elucidation of the cellular interactions that target and control the sheddase activity of ADAMs, and mediate their effects on intracellular signaling and sorting processes is needed to better understand and to therapeutically target the contribution of ADAMs to the pathogenesis of cancer and other major diseases.

## Supporting Information

S1 Table Customized SH3-phage libraries used in follow-up screens. Several different reduction libraries lacking an increasing number of already identified SH3 domains were constructed and used as indicated to reveal additional ADAM interactors in the absence of their most preferred SH3 binding partners.
(PDF)
