# Supplementary material for: Preferred SH3 Domain Partners of ADAM Metalloproteases Include Shared and ADAM-Specific SH3 Interactions
Source: PLoS One. 2015 Mar 31;10(3):e0121301. doi: 10.1371/journal.pone.0121301 (PMC4380453; doi:10.1371/journal.pone.0121301)
Supplement: S1 Table — Several different reduction libraries lacking an increasing number of already identified SH3 domains were constructed and used as indicated to reveal additional ADAM interactors in the absence of their most preferred SH3 binding partners. (PDF) [file pone.0121301.s001.pdf]

Library	SH3-domains removed from library	ADAMs screened
RL-1	SNX33	ADAM9, -12, -15
RL-2	Tks5(I)	ADAM19
RL-3	SNX33	ADAM15
RL-4	Tks5(I)	ADAM19
RL-5	SNX33, SNX9	ADAM9
RL-6	TOCA1, SNX33	ADAM8
RL-7	SNX33, SNX9, Tks5(V)	ADAM15
RL-8	Tks5(I), NCF1(I)	ADAM19
RL-9	TOCA1, SNX33, SNX9	ADAM8
RL-10	TOCA1, SNX33, CIP4, Tec, Src	ADAM8
RL-11	SNX33, SNX9, SNX18, Src	ADAM9
RL-12	Tec, SNX33	ADAM10
RL-13	Src, nephrocystin	ADAM12
RL-14	SNX33, Tks5(V), Src	ADAM15
RL-15	Tks5(I), NCF1(I), Tks5(V), RIMBP1(III), Myosin VIIA, Src	ADAM19
RL-16	TOCA1, SNX33, SNX9, CIP4, Tec, Src	ADAM8
RL-17	SNX33, SNX9, SNX18, Tec	ADAM9
RL-18	Src, nephrocystin	ADAM12
RL-19	SNX33, Tks5(V), Src	ADAM15
RL-20	Tks5(I), NCF1(I), Tks5(V), RIMBP1(III), Eps8L1, Src	ADAM19
RL-22	Src, nephrocystin, Tks5(V), Lyn	ADAM12
RL-23	SNX33, Tks5(V), Src, Tec, nephrocystin, NCF1, Tks5(I), Lyn	ADAM15
RL-24	TOCA1, SNX33, SNX9, CIP4, Tec, Src, OSTF1, NCF1(I)	ADAM8
RL-25	SNX33, SNX9, SNX18, Tec, NCF1(I), Lyn, p85α	ADAM9
RL-26	Src, nephrocystin, Tks5(V), Lyn, Hck, SNX33, SNX9, AHI1	ADAM12
RL-27	Src, nephrocystin, Tks5(V), Lyn, Hck, SNX33, SNX9, AHI1, OSTF1	ADAM12
RL-28	SNX33, Tks5(V), Src, Tec, nephrocystin, Tks5(I), Lyn, SNX9, Hck, p85α, OSTF1	ADAM15
RL-29	Tks5(I), Src, NCF1(I), RIMBP1(III), Tec, Lyn, Tks5(V), Eps8L1, p85α, SNX33, nephrocystin, SNX9, TOCA1	ADAM19
